# Development and evaluation of an online tool for management of overweight children in primary care: a pilot study

**DOI:** 10.1136/bmjopen-2014-007326

**Published:** 2015-06-12

**Authors:** Min Hae Park, Áine Skow, Dewi Ismajani Puradiredja, Anna Lucas, Hayley Syrad, Ulla Sovio, Billy White, Anthony S Kessel, Barry Taylor, Sonia Saxena, Russell M Viner, Sanjay Kinra

**Affiliations:** 1 Department of Non-communicable Disease Epidemiology, London School of Hygiene & Tropical Medicine, London, UK; 2 Department of Population Health, London School of Hygiene & Tropical Medicine, London, UK; 3 Faculty of Life Sciences and Computing, School of Psychology, London Metropolitan University, London, UK; 4 Department of Epidemiology and Public Health, Health Behaviour Research Centre, University College London, London, UK; 5 Department of Obstetrics & Gynaecology, University of Cambridge, Cambridge, UK; 6 UCL Institute of Child Health, University College London, London, UK; 7 Faculty of Public Health and Policy, London School of Hygiene & Tropical Medicine, London, UK; 8 Department of Women's & Children's Health, Paediatrics & Child Health, University of Otago, Dunedin, New Zealand; 9 Department of Primary Care and Public Health, School of Public Health, Imperial College London, London, UK

**Keywords:** Childhood obesity, Online tool, Lifestyle behaviours, General practice, Family practice

## Abstract

**Objective:**

To explore the acceptability of implementing an online tool for the assessment and management of childhood obesity (Computer-Assisted Treatment of CHildren, CATCH) in primary care.

**Design and setting:**

An uncontrolled pilot study with integral process evaluation conducted at three general practices in northwest London, UK (November 2012–April 2013).

**Participants:**

Families with concerns about excess weight in a child aged 5–18 years (n=14 children).

**Intervention:**

Families had a consultation with a doctor or nurse using CATCH, which assessed child weight status, cardiometabolic risk and risk of emotional and behavioural difficulties and provided personalised lifestyle advice. Families and practitioners completed questionnaires to assess the acceptability and usefulness of the consultation, and participated in semistructured interviews which explored user experiences.

**Outcome measures:**

The primary outcome was family satisfaction with the tool-assisted consultation. Secondary outcomes were practitioners’ satisfaction, and acceptability and usefulness of the intervention to families and practitioners.

**Results:**

The majority of families (86%, n=12) and all practitioners (n=4) were satisfied with the consultation. Participants reported that the tool was easy to use, the personalised lifestyle advice useful and the use of visual aids beneficial. Families and practitioners identified a need for practical, structured support for weight management following the consultation.

**Conclusions:**

The results of this pilot study indicate that an online tool for assessment and management of childhood obesity can be implemented in primary care, and is acceptable to patients, families and practitioners. Further development and evaluation of the tool is warranted.

**Strengths and limitations of this study**

- Primary care practitioners in the UK report that effective screening and management of childhood obesity is limited by time constraints and lack of training.
- We developed an online tool that enables practitioners to assess child weight status, estimate weight-related health risk and provide personalised evidence-based advice to families.
- In this pilot study, the tool was implemented in three general practice clinics by doctors and nurses and was shown to be acceptable and helpful to families, while practitioners found the tool useful, easy to use and time-saving. Further work is needed to assess the feasibility of using the tool within the time constraints of typical primary care consultations.
- The generalisability of findings is limited by its small sample, which is not representative of the wider target population. There is a need to explore the usefulness and acceptability of the tool among different populations and practitioners, and in scenarios in which childhood obesity is not self-referred.

## Introduction

In the UK, more than one in three children aged 10–11 years are overweight or obese.^1^ Excess weight in childhood is associated with increased risk of adult obesity and its comorbidities,^2^ ^3^ as well as health problems in childhood.^4^ ^5^ For most families, initial assessment of childhood obesity and access to treatment will be through primary care. However, primary care practitioners in the UK report that effective screening and management is limited by time constraints, insufficient training and sensitivity of raising weight-related issues.^6–8^ Formal training in child anthropometry is uncommon in general practice,^9^ ^10^ and practitioners report a lack of expertise to treat childhood obesity.^8^

Several studies indicate that the use of technology could improve childhood obesity treatment in primary care by supporting practitioners to identify and manage cases.^11–15^ We developed an evidence-based online tool for the assessment and management of childhood obesity: Computer-Assisted Treatment of CHildhood overweight (CATCH). CATCH incorporates national clinical guidelines for the assessment and management of childhood obesity^16^ and risk stratification based on analysis of population-based data,^17^ and enables practitioners to provide personalised, evidence-based advice to families. We present data from a pilot study which implemented CATCH in primary care, and assessed families’ and health professionals’ satisfaction with consultations using the tool.

## Methods

### Study design and recruitment

We conducted an uncontrolled pilot intervention study with an integral process evaluation at three general practices in northwest London. Practices and clinicians were identified through the Primary Care Research Network. We approached four practices to participate in the study, of which three agreed; at the three participating clinics, all practitioners who were invited to take part in the study agreed. Practices mailed letters to the parents of all registered children aged 5–18 years (∼2600 children), inviting them to participate in the study.

### Participants

Families were eligible to participate if they presented at one of the practices during the study period (November 2012–April 2013) with concerns about excess weight in a child aged 5–18 years. The child did not have to be overweight or obese to take part. Families in which the parents or child could not read English, and families of children receiving care for weight management were excluded.

### Description of intervention

The intervention was a single consultation with a general practice doctor (n=2) or nurse (n=2) using CATCH, a secure web-based application that is accessed through standard internet browsers.^18^ CATCH was designed to follow the standard flow of a clinical consultation, and the content and design of the tool was developed in consultation with general practitioners (GPs) and practice nurses unaffiliated with the research team. Throughout the development process we conducted individual and group meetings with these clinicians, including discussions to determine current practices and consultation content, and used their feedback to develop and make adjustments to the tool as necessary. The functionality and usability of the tool were assessed during the web development stage by piloting a paper-based version of the application.

CATCH guides a practitioner through a consultation involving three main steps; screen shots of the tool are provided in online supplementary material 1:

1. Calculation of the child's body mass index (BMI) centile and weight status using measured weight and height (assessed by the practitioner during the consultation), and weight status displayed on a BMI chart (overweight defined as BMI ≥91st centile of the UK90 reference,^19^ obese as BMI ≥98th centile, in accordance with clinical guidelines^16^).
2. Risk assessment: risk estimation and stratification to identify children at increased risk of a weight-related comorbidity based on brief sociodemographical information collected during the consultation, and recommendation for further assessment or referral where relevant. Comorbidities were selected based on a systematic review which identified two broad categories of childhood obesity comorbidities: cardiovascular risk factors and mental health conditions. Based on analysis of data from two large population-based cohorts of UK children (the Avon Longitudinal Study of Parents and Children (ALSPAC)^20^ and Research with East London Adolescents—Community Health Survey (RELACHS)^21^), we developed two risk algorithms: one to estimate a child's current risk of having one or more cardiovascular abnormalities (fasting glucose ≥5.6 mmol/L,^22^ LDL-cholesterol ≥2.85 mmol/L^23^ or systolic or diastolic blood pressure ≥95th centile for age, sex and height^24^), and one to estimate a child's current risk of emotional and behavioural difficulties (strengths and difficulties questionnaire score >17.5 indicates raised probability of psychiatric disorder).^25^ ^26^ The risk estimation models were developed using logistic regression methods to assess the cross-sectional associations between exposures and outcomes in overweight and obese children. Demographic, anthropometric and family characteristics were added to each model in stepwise fashion, and the fit of the model was assessed after the inclusion of each variable using area under the curve (AUC) estimated from the receiver operating characteristic curve. Exposures that were associated with the outcome (p<0.05) and resulted in an improvement in AUC were retained in the final model. The only variable retained in the final cardiovascular risk estimation model was a measure of BMI adjusted for age, sex and ethnicity (an ethnicity-adjusted BMI z-score; p<0.001). For emotional and behavioural difficulties, the variables included in the final model were bullying status (p=0.001), victimisation status (p=0.003) and hours of sedentary behaviour (p=0.002). For each outcome, risk scores were classified as low, medium or high: the cut-off that would classify a child as ‘High risk’ was chosen based on the prevalence of the outcome among overweight children in the relevant data set. For example, if the prevalence of the outcome among overweight children was 10%, then the ‘High risk’ cut-off was set so that the proportion of overweight children with scores that exceed the cut-off would be 10%. The cut-off for ‘Low risk’ was chosen to maximise the negative predictive value of the model. When tested in the databases from which they were derived, both models had high sensitivity when used to identify low-risk children (85–89%). When used to identify high-risk children, sensitivity was low: 23.3% for emotional and behavioural difficulties; 52.7% for cardiovascular risk factors. However, when compared to the performance of similar algorithms, for example, models to detect children at risk of glucose intolerance^27^ or atherosclerotic lesions,^28^ our specificity figures (59–95%) were comparable and our positive predictive values (36–53%) were higher (see online supplementary material 2).
3. Lifestyle assessment: generation of printable, personalised lifestyle advice based on patient-reported information about diet, physical activity and sleep patterns. These lifestyle behaviours were selected based on a literature review of modifiable behaviours associated with childhood overweight, and analysis of data from the ALSPAC cohort to identify specific behaviour variables associated with BMI among overweight and obese children. For each lifestyle variable, we created an algorithm to compare patient-reported information with recommended levels for these behaviours.^29^ If a child meets the recommended level, encouraging feedback is provided; if the recommended level is not met, the behaviour is identified as an area needing improvement and lifestyle modification suggestions are provided.

### Outcomes

The primary outcome was family satisfaction with the tool-assisted consultation. Secondary outcomes were practitioners’ satisfaction, and acceptability and usefulness of the intervention to families and practitioners.

### Data collection

Practitioners were interviewed before the trial period to collect information on childhood obesity care before implementation of the tool, including the number of families attending with weight concerns, normal practice in a consultation with an overweight child, treatment options and barriers to management.

After the consultation, parents completed a self-administered 11-item questionnaire (see online supplementary material 3). The questionnaire asked about satisfaction and acceptability of the tool. Parents were also asked about the usefulness of the risk assessment and the personalised lifestyle advice, and the quality of care they had received. Practitioners completed an online questionnaire after each consultation. They rated the usefulness and ease of using the tool, and were asked whether the tool saved time, improved their ability to provide care and whether they would recommend it to other healthcare professionals. All questionnaire items were scored using four or five point Likert scales.

All participating families and practitioners were invited to take part in the semistructured, face-to-face interviews which explored user experiences with the tool. Interviews with practitioners lasted on average 12 min (range 7–36 min), and were conducted at the GP surgeries. Interviews with families lasted 12.5 min (9–17 min) and took place in the participants’ homes. Interviewers used open questions and probes to explore the main themes in the questionnaires (acceptability, satisfaction, usefulness) and to capture emergent themes. Interview topic guides are provided in online supplementary material 4. All interviews were audio recorded and transcribed verbatim.

### Analysis

Questionnaire data were summarised using frequency counts and proportions. Family satisfaction was defined as the proportion of parents who were ‘Very satisfied’ or ‘Extremely satisfied’, and acceptability as the proportion who were ‘Comfortable’ or ‘Very comfortable’ with the consultation. Practitioners’ responses from their first completed questionnaire were analysed. We report the proportions of practitioners who found the tool useful, were satisfied with the consultation and found the tool easy to use.

Qualitative data were analysed (by author DIP) using NVIVO software V.10.0 (QSR International, Southport, UK). The Framework analysis approach was adopted,^30^ using both deductive and inductive methods. Transcripts were read and re-read to identify a priori and emerging themes to be used as coding categories. Matrix-based thematic frameworks were developed (one for practitioners and one for families), into which the textual data were indexed and summarised by frequency for the purpose of interpretation.

## Results

Fifteen young people attended the practices with concerns about excess weight and were assessed for eligibility. Of these, one did not meet the inclusion criteria (aged >18 years) and therefore, was not invited to participate in the study. In total, 14 children from 12 families (including two sibling pairs) received the intervention. A family questionnaire was completed after each consultation (n=14), and 9 families (n=11 children) were interviewed. One family did not consent to be interviewed, another could not be contacted and another missed two scheduled interviews and was assumed to no longer wish to participate. With the exception of one interview in which both parents participated, interviews were conducted in the presence of the mother and child; in both families with sibling pair participants, only one child was present during the interview. The characteristics of participating children are shown in table 1.

**Table 1:** Characteristics of children that received a computer-assisted consultation for concerns about excess weight at one of three general practices in northwest London during the pilot study period (n=14)

Characteristic	Mean (±SD) or % (n)
Age (years)	10.7 (±2.6)
Sex—female	50% (7)
Ethnicity	
White	7.1% (1)
Asian	64.3% (9)
Black	28.6% (4)
Height (cm)	148.6 (±14.3)
Weight (kg)	54.0 (±12.5)
BMI	24.1 (±2.3)
BMI Z-score	2.25 (±0.6)
Weight status*	
Healthy weight	7.1% (1)
Overweight	25.7% (5)
Obese	57.1% (8)

*Cut-offs at 91st and 98th centiles of the UK 1990 reference population to define overweight and obesity.

BMI, body mass index.

### Baseline interviews with practitioners

Five practitioners were interviewed before the trial period (3 doctors, 2 nurses; one doctor who was interviewed at baseline did not take part in intervention delivery). Respondents reported that they saw very few children presenting with overweight or obesity as a primary health concern (up to 25 children each year). All practitioners reported that they did not normally measure BMI or conduct other tests, and they would give general advice about diet and physical activity rather than any specific treatment. However, uptake of lifestyle advice was seen to be poor, and none of the practitioners routinely engaged in proactive follow-up due to the lack of response from patients.

### Family questionnaire

#### Satisfaction and acceptability

Among respondents to the family questionnaire, 86% (n=12 out of 14) were satisfied with the tool-aided consultation. The remaining respondents were ‘somewhat satisfied’. All parents reported that they and their child felt comfortable with the consultation. All parents were comfortable being asked about their child's lifestyle and medical history, but one parent was ‘slightly uncomfortable’ when asked about whether their child had been teased or bullied.

#### Usefulness and quality of care

The majority of respondents (79%, n=11) found it useful to receive personalised feedback on weight management; 21% (n=3) found it ‘somewhat useful’. All parents reported that their child was: (1) asked questions about his/her health habits, (2) helped to set goals to improve lifestyle, and (3) given a copy of their treatment plan. All parents agreed that they were treated with care and concern, that their child's care was well organised and that they had confidence and trust in their practitioner.

### Family interviews

Table 2 shows the frequency of main themes in the analytical framework. In line with the questionnaire responses, family interviews revealed that experiences with CATCH were overall positive. Parents described the consultation as informative, non-judgmental and non-intrusive.

I think the questions were very good because there are a lot of things that we were doing and we didn't know that it was good or bad. It was good. The whole consultation was very beneficial.

Mother of boys aged 10 (obese) and 12 (overweight)

**Table 2:** Themes in the analytical framework for qualitative interviews with families (n=9 families, 11 children) and the number of participants discussing them

Themes	Number of participants
Reasons for consultation	
Parents’ concern about child's weight	6
Child's concern about her/his weight	1
GP/nurse advised them to	2
Expectation of free practical support (with weight issues)	1
Acceptability	
Parents responded positively to tool-based consultation (eg, with interest)	10
Children responded with apprehension to tool's outputs	1
Problems with the risk assessment feature	0
Reasons for perceived usefulness	
Generally useful	12
Informative	5
Advice is instructive	5
Impact of lifestyle advice	
Dietary changes	4
More physical activity	3
Weight loss	1
Go to sleep earlier	1
Lifestyle changes for whole family	2
Satisfaction	
Overall satisfied	12
Would recommend it to others	8
Reasons for (Dis-)satisfaction	
Generally satisfied	12
Revelatory: ‘Wake-up call’	1
Questions format is sermonic in places	1
Delivery of advice could have been more assertive	1
Recommendations	
Have follow-up consultations/practical support (including monthly weight targets)	5
Address psychological issues	1
More tailored question format	1

GP, general practitioner.

All interviewees found the tool's outputs useful. Even though most parents had been concerned about their child's weight previously, several referred to the consultation as a ‘wake-up call’ that alerted them to the severity of the problem.

[…] I would know that [my daughter was] putting on weight but I would not say, I didn't expect that it was as bad as it was and especially when they weighed her and they found she was 61 [kg] when she's nine. It was a wake-up call. It was good.

Mother of girl aged 9, obese

However, two parents described that the results of the consultation had generated some anxiety in their children:

[…] when she saw what was on the computer that she was on the red line and they explained to her what it means for her health […] she really [got] scared.

Mother of girl aged 9, obese

[My son] was a little bit worried [when hearing the results of the consultation] like, ‘Oh what's going to happen next?’ [But in the end] everything was fine.

Mother of boy aged 9, obese

All of the respondents found the lifestyle advice informative and instructive. In particular, specific advice on diet was highlighted as being useful:

[I found the lifestyle advice] very, very helpful because I was buying some stuff like I wanted them to eat breakfast, they eat breakfast every morning but I was buying them wrong breakfast, the cereals. […] You know the sugary stuff so now […] I've cut down on those things and the drinks that I was buying, I thought they are orange juice and stuff but [it's] not 100% juice…

Mother of boys aged 10 (obese) and 12 (overweight)

The use of visual aids, such as the BMI chart and the printed lifestyle advice, was described as reinforcing the advice given:

It just confirmed everything that we already knew but you know when it's on paper it's sort of a bit more…I wouldn't say serious but it's a bit more in your face.

Mother of boy aged 7, overweight

Five interviewees said they had made lifestyle changes following the tool-based consultation. In at least two cases, lifestyle changes had been introduced for the whole family. A theme that emerged was the need for practical support following the consultation. Five parents suggested that follow-up appointments for monitoring and guidance on weight management would be beneficial.

I would probably say that now, as a stepping forward thing, although the study thing may be over, maybe to follow-up and do like a plan on how to maintain or reduce the weight or some kind of thing like that. […] To be honest, I don't mind doing it for myself but then there's no monitoring or anything like that through the GP which might be beneficial for them as well so they can keep an eye on whether we're doing it correctly and there's no other side effects or anything like that.

Mother of boy aged 7, overweight

Most parents seemed to be unaware of local services to support weight management. One mother (boy aged 9, obese) had joined a group to learn more about healthy nutrition and cooking, and suggested that more services like this would be helpful.

### Practitioner questionnaire

#### Satisfaction

All practitioners (n=4) were satisfied with the consultation. Three practitioners indicated that they would recommend the tool to other health professionals; one was not sure whether they would recommend it.

#### Usefulness and ease of using the tool

Two respondents reported that the tool was useful during the consultation; two found it ‘somewhat useful’. All practitioners reported that the tool was easy to use. We attempted to record the duration of each consultation by collecting data on the start and end time of each online session of the tool, but reliable data were not available due to issues with time recordings, such as practitioners opening an online session before the consultation and not closing the sessions afterwards. However, three respondents agreed that using the tool saved them time; one respondent ‘slightly agreed’. The same three respondents agreed that using the tool improved their ability to care for the child.

#### Quality of care

All respondents reported that they felt confident of their skills and knowledge during the consultation. All practitioners felt that they provided the patient with appropriate treatment, had contributed to the patient's well-being and had provided well-organised care.

### Practitioner interviews

The analytical framework used for practitioner interviews is shown in table 3. Overall, practitioners described satisfaction with the tool. A theme that emerged was that the tool could enhance the impact of practitioners’ advice by adding authority to the message being communicated:

[…] I thought that was really good. We talked about it all the time. And because patients can take it away with them, it is quite good for them. I literally go over what the [lifestyle advice] told them: what you should eat [etc.] And they got something in writing from the computer. They see ‘I'm not just saying that’…that [there is truth] to it. (nurse, practice 3)

**Table 3:** Follow-up themes in the analytical framework for qualitative interviews with primary care practitioners (n=4) and the number of participants discussing them

Themes	Number of participants
Feasibility	
Overall electronic consultation delivery: Unproblematic	4
Output interpretation: unproblematic	4
*Acceptability*	
Patients’ overall response to tool	
Patients responded positively to tool-based consultation (eg, with interest)	3
Children responded with indifference to tool's outputs	1
Left wanting for more detailed advice/tangible/practical support	2
Parents showed more concern than children	1
Patients’ responses to individual features	
Found BMI chart revelatory	2
Found lifestyle advice helpful/informative	3
Found lifestyle advice sermonic	1
Parents seemed uncomfortable with emotional risk assessment questions	2
Reasons for (Dis-)satisfaction	
Generally satisfied	4
Provides BMI chart tailored to children	2
Provides print outs with lifestyle advice	3
Gives opportunity to discuss weight issues and lifestyle choices	3
Provides enquiries not normally covered in routine consultations	2
Provides ‘authoritative’ information (empowering to staff and parents)	1
Question format can be perceived as overly sermonic	1
Recommendations	
Integrate tool into clinical software system	4
Have follow-up consultations/practical support (including monthly weight targets)	3
Redesign lifestyle advice to speak to youth	1
More concise question format	1

BMI, body mass index.

This ‘empowering effect’ of the tool was also discussed by one respondent in relation to supporting parents in convincing their children of the importance of lifestyle changes.

All practitioners found the tool straightforward to use, although one respondent noted that it required some practice (nurse, practice 3). None of the practitioners reported having problems interpreting the tool's outputs.

One respondent (doctor, practice 1) felt that children reacted to the consultation with indifference, but other interviewees felt that patients reacted positively. The BMI chart and lifestyle advice were highlighted as generating the strongest responses from children and parents.

I think for some people they probably underestimated the time that their kids were spending watching TV or playing games, or computer games or whatever. So that was good. The thing about sleep was good. How much sleep that they need on average, was good. […] The other thing was about fruit juices. Sugar. I think that was a revelation to a lot of people. I think, you know, there was nothing negative about it [the tool]. (doctor, practice 2)

It was also suggested that the chart could be used as positive reinforcement for patients with BMI in the healthy range.

[…] in fact, we had one youngster who came along and [the BMI chart showed that] he was perfectly healthy […] and actually, it was good for them [to hear that] ‘Well, actually what you're doing is fine. What you're doing is right, correct. You've a healthy weight, you're eating properly’. It was a good opportunity to say to them: well done! (nurse, practice 2)

Although none of the practitioners described serious problems with the tool, two respondents described that it can be uncomfortable to broach the subject of a child's weight-related health risk with parents:

[The risk assessment component] was tricky as far as the parent's concerned. […] The parents found it uncomfortable. Maybe it opened something up for them to say, you know…Is this going on [with my child]? Am I not being told? (nurse, practice 2)

All four respondents were in agreement that the tool (or a version of it) would be something they would continue to use in the future and would like to see integrated into their clinical software system. In particular, the child-specific BMI chart was seen to be desirable, as current tools on the system only allow calculation of adult weight status.

Three of the four respondents suggested that the tool-based consultations would benefit from the addition of more tangible support, such as a structured programme of activities and follow-up consultations to monitor weight management.

## Discussion

Recent years have seen an increase in the development of electronic tools and other forms of information technology for healthcare. A handful of tools that aim to facilitate the treatment of overweight children in primary care have been identified, including modified electronic medical records (EMRs) for assessment of BMI centile and weight status,^12^ an online tool for assessing secondary causes of obesity and serious comorbidities in obese children^13^ and a computerised decision-support tool to assess weight status in children and provide semipersonalised lifestyle advice.^14^ Among these, CATCH is distinct in that it performs both risk prediction to facilitate referral of comorbidities and provides personalised lifestyle advice.

The findings from our evaluation study provide preliminary evidence that CATCH can feasibly be implemented in UK primary care settings. Overall, family and practitioner experiences with the tool-assisted consultations were positive. The majority of families were satisfied with their consultation, and found the personalised weight management advice useful, particularly in relation to diet. Practitioners reported that they did not routinely assess or treat childhood obesity, preintervention, indicating a need for greater focus on childhood obesity in primary care; they all found the tool easy to use, and most reported that they would recommend it to other health professionals. Further development to integrate the tool into current clinical software systems may be desirable. Practitioners also reported that the tool saved them time, but further work is needed to assess consultation duration using CATCH, and to establish the feasibility of using the tool within the time constraints encountered in typical primary settings. A common theme that was identified by families and practitioners was the increased impact of practitioners’ advice due to the use of visual aids and the perceived authority of the tool. This is consistent with previous studies which have indicated that appropriate visuals can help individuals to understand health risks.^31^ Additionally, personalised estimates of risk have been shown to be effective tools for increasing knowledge and improving risk perception among patients.^32^ Most parents made lifestyle changes for their child following the tool-based consultation, and in some cases extended these changes to the whole family, pointing to potential health benefits of the tool beyond those for the overweight child. Reliable data on the duration of each consultation were not available, but clinicians reported that the tool saved them time.

Parents and practitioners highlighted the need for follow-up and structured support for children identified as overweight or obese. Follow-up care is likely to be an important factor in weight management success; therefore, adequate care pathways need to be put in place before assessment tools are implemented on a large scale. Further work is needed to establish the impact of this type of brief intervention on health outcomes, including lifestyle behaviours and weight loss.^15^ Concerns about the tool were related to anxiety in children, and practitioners feeling uncomfortable discussing weight-related health problems with parents; these point to the importance of training for practitioners to deliver sensitive information in a supportive and non-judgmental manner. Despite these concerns, all parents reported that their child felt comfortable with the consultation, and most practitioners felt that the tool improved their ability to care for the child.

The generalisability of findings from this pilot study is limited by the small number of study sites and small sample, which is not representative of the wider target population. In particular, due to the ethnically diverse population in northwest London, the majority of participants in our study were from Asian or black ethnic groups (in the boroughs of Harrow and Brent, around two-thirds of the population are from Black and Minority Ethnic communities, predominantly South Asian groups^33^). Children from these ethnic groups are at increased risk of obesity^34^ and associated health problems^35^ and may, therefore, be priority groups for obesity interventions. However, parents who participated in the study are likely to be those who are engaged with weight-related health issues; experience of the tool-based consultation may be different if administered to families with less interest in these issues. Similarly, the practitioners that participated in this study were identified through the Primary Care Research Network and are, therefore, not likely to be representative of all practitioners; the success of tool-based consultations may vary if delivered by clinicians with different interests. There is a need to explore the usefulness and acceptability of CATCH in scenarios in which childhood obesity is not self-referred, for example, in cases where a doctor or nurse identifies a weight problem and conducts a consultation using the tool. We were unable to assess this in the present study due to the ethical review committee's decision to forbid opportunistic recruitment (active targeting of overweight children), and we were required to use blanket mail-outs to recruit participants. Given that a low proportion of parents recognise overweight or obesity in their children,^36^ the restrictions on active recruitment may explain the relatively low number of families attending the clinics, although uptake could be considered to be reasonable given that at baseline each practitioner reported seeing fewer than 25 children per year presenting with overweight or obesity as a primary health concern. There remains a need to assess the acceptability and experience of using the tool among different patient populations.

Despite these limitations, this evaluation has identified key themes relating to the acceptability and usefulness of an online tool for the assessment and management of overweight children in primary care, and these provide the basis for further intervention development and evaluation. There are plans to validate and develop the risk prediction models used in the tool in larger samples of overweight and obese young people, and to assess the feasibility and acceptability of the tool in a larger evaluation study.
